# Rural-Urban Variation in the Association of Adolescent Violence and Handgun Carrying in the United States, 2002-2019

**DOI:** 10.1001/jamanetworkopen.2023.1153

**Published:** 2023-02-28

**Authors:** Julia P. Schleimer, Emma Gause, Kimberly Dalve, Alice Ellyson, Ali Rowhani-Rahbar

**Affiliations:** 1 Department of Epidemiology, University of Washington, Seattle; 2 Firearm Injury and Policy Research Program, University of Washington, Seattle; 3 Department of Pediatrics, University of Washington, Seattle; 4 Center for Child Health, Behavior, and Development, Seattle Children’s Research Institute, Seattle, Washington

## Abstract

**Importance:**

Adolescent handgun carrying is associated with increased risk of firearm-related violence. Most evidence on adolescent handgun carrying is from urban areas, but these findings may not generalize to rural areas.

**Objective:**

To examine differences in associations of adolescent interpersonal violence with handgun carrying across the rural-urban continuum.

**Design, Setting, and Participants:**

This cross-sectional study used nationally representative data from the US National Survey on Drug Use and Health among adolescents aged 12 to 17 years from 2002 to 2019 to estimate time-varying prevalence ratios (PRs) and prevalence differences (PDs) between interpersonal violence and handgun carrying across the rural-urban continuum. Analyses were conducted in April to July 2022.

**Exposures:**

Any past-year serious fighting, group fighting, and attacking with intent to harm.

**Main Outcomes and Measures:**

Any past-year handgun carrying. Associations were estimated within county rural-urban strata using the US Department of Agriculture’s Rural-Urban Continuum Codes.

**Results:**

In each year, the sample included a weighted count of almost 25 million adolescents, with 50.9% (95% CI, 50.2%-51.6%) males and 24.7% (95% CI, 23.8%-25.6%) Hispanic adolescents, 13.5% (95% CI, 12.8%-14.2%) non-Hispanic Black adolescents, and 51.8% (95% CI, 50.8%-52.8%) non-Hispanic White adolescents in 2019. More rural counties had less racial and ethnic diversity. For example, 81.1% (95% CI, 75.9%-85.4%) of adolescents were non-Hispanic White in the most rural counties vs 43.1% (95% CI, 41.7%-44.6%) of adolescents were non-Hispanic White in the most urban counties in 2019. Adolescent handgun carrying increased over time, with the largest increases in the most rural counties, where the prevalence of adolescent handgun carrying increased from 5.2% (95% CI, 3.8%-7.0%) in 2003 to 12.4% (95% CI, 8.9%-16.9%) in 2019. PRs for the association of violence and handgun carrying were greater in more urban counties. For example, in the most urban counties in 2019, adolescents involved in a group fight had 3.7 (95% CI, 2.9-4.8) times the prevalence of handgun carrying vs those not involved in a group fight; this PR was 3.1 (95% CI, 1.6-5.6) in the most rural counties. PDs were similar and, in some cases, larger in rural areas. For example, in the most urban counties in 2019, handgun carrying prevalence was 7.5% (95% CI, 5.7%-9.5%) higher among adolescents who were involved in a group fight compared with those who were not; this PD was 21.8% (95% CI, 8.2%-37.8%) in the most rural counties, where handgun carrying was more common.

**Conclusions and Relevance:**

This cross-sectional study found that associations of interpersonal violence with handgun carrying were stronger in relative terms in urban areas than in rural areas; however, a higher percentage of rural than urban adolescents carried handguns, resulting in a greater absolute prevalence of handgun carrying associated with violence in rural areas than in urban areas. These findings suggest opportunities for preventing handgun carrying–related harms may differ between rural and urban communities.

## Key Points

**Question:**

Do associations between adolescent participation in violence and handgun carrying vary across the rural-urban continuum in the United States?

**Findings:**

In this cross-sectional study using survey data from 2002 to 2019 with a weighted count of nearly 25 million adolescents per year, adolescent handgun carrying was most common in the most rural counties. The greater overall prevalence of handgun carrying in rural areas was associated with a higher absolute prevalence of handgun carrying associated with adolescent violence in rural areas than in urban areas.

**Meaning:**

These findings suggest that adolescent participation in violence was associated with handgun carrying across the rural-urban continuum; however, opportunities for preventing handgun carrying–related harms may differ between rural and urban communities.

## Teaser

This cross-sectional study examines differences in associations of adolescent interpersonal violence with handgun carrying across the rural-urban continuum.

## Introduction

Firearm-related violence among young people is a pressing public health problem in the US.^1^ In 2020, suicide and homicide were leading causes of death among adolescents aged 12 to 17 years, with firearms involved in 44% of suicide deaths and 91% of homicide deaths.^2^

The characteristics of adolescent firearm-related injury vary across the rural-urban continuum. The number of firearm-related deaths among adolescents aged 12 to 17 years is generally higher in urban areas, but recently (2016-2020), the rate was higher in noncore nonmetropolitan counties (7.7 injuries per 100 000 population) than large central metropolitan counties (7.3 injuries per 100 000 population), with counties classified per the National Center for Health Statistics.^3,4^ Youth firearm-related violence has often been viewed as a problem of urban crime,^5,6^ perhaps because adolescent firearm-related homicide rates are higher in urban vs rural areas.^3,7^ However, when considering nonfatal and fatal firearm-related injuries together, rates of interpersonal injuries are greater than self-inflicted injuries among rural youth and young adults.^8^ Furthermore, rural and urban adolescents alike commonly experience or witness multiple forms of interpersonal violence, eg, being threatened or attacked.^9,10,11^ Research is needed on adolescent firearm-related harms of all types across the entire rural-urban continuum.

The social, cultural, and environmental contexts of rural areas are, in many ways, distinct from urban areas. These differences exist across multiple socioecological levels and likely shape adolescents’ exposure to firearms and risk factors for adolescent firearm-related injury.^12,13,14,15^ Firearm ownership is more common in rural areas,^16,17^ and, at the individual level, rural adolescents are more likely than their urban peers to report firearm access and use.^18,19,20^ At family and peer levels, rural adolescents are likely to be exposed to firearms at early ages, and many have experience with formal firearm training.^16,21,22^ At the community level, especially in the most rural parts of the US where agriculture, forestry, and other resource-based industries are prominent,^23^ young people might use firearms for work or recreation. In fact, while adolescents are generally not legally allowed access to firearms, federal law permits handgun possession and use by individuals younger than 18 years for employment, ranching or farming, and target practice, among other limited purposes.^24^

Notwithstanding potential firearm use for recreation, sport, or work, adolescent firearm access is associated with increased risk of firearm-related harms.^25,26,27,28,29^ Adolescent handgun carrying in particular is an important precursor to firearm-related injury, especially assault,^30,31^ although handguns are commonly used in all types of adolescent firearm-related injury.^32,33^ Even instances of adolescent handgun carrying that do not result in firearm injury could increase the threat of violence and undermine community members’ perceptions of safety. Therefore, adolescent handgun carrying is considered an important point of intervention to prevent firearm-related harm.

Handgun carrying is more common among adolescents in rural than urban communities.^34^ From 2002 to 2019, 5.1% of adolescents ages 12 to 17 years in rural counties (ie, <20 000 urban population) reported past-year handgun carrying, compared with 3.9% in small metropolitan counties (ie, 20 000-1 000 000 urban population) and 3.1% in large metropolitan counties (ie, >1 000 000 urban population).^34^ Increases in adolescent handgun carrying during the last decade were most pronounced in rural counties, suggesting changes in the nature or prevalence of factors that contribute to rural adolescent handgun carrying.^34^

Despite the high and increasing prevalence of adolescent handgun carrying in rural areas, researchers have, until recently,^18,22,34,35,36^ almost exclusively studied handgun carrying among adolescents in urban areas.^30^ In these settings, antisocial and aggressive behavior (eg, fighting) are associated with handgun carrying.^30^ Therefore, interventions to prevent adolescent handgun carrying and associated harms have typically focused on adolescents at heightened risk for violence (eg, deterrence-based strategies rooted in criminology).^37^ However, given distinct contexts of rural communities (eg, greater household firearm access and firearm use for recreation and work), research findings and prevention strategies from urban settings might not generalize to rural ones. Indeed, a 2020 commentary by Culyba^6^ called on researchers to ask “Is handgun carrying in rural settings associated with the same constellation of health risk behaviors observed in urban settings?” We have especially limited knowledge of adolescent handgun carrying across the spectrum of rurality. Of the few studies to examine rural-urban variation in adolescent handgun carrying, most have grouped communities into broad rural and urban categories,^34,38,39^ obscuring potential heterogeneity within them. This is an important limitation, since rural-urban status is best conceptualized as a continuum^40^; studies that mask this variation may also mask the unique contexts, experiences, and needs of communities.

We used nationally representative cross-sectional survey data among adolescents aged 12 to 17 years in the US from 2002 to 2019 to examine relative and absolute time-varying associations of adolescent interpersonal violence and handgun carrying across 6 county-level rural-urban categories. Relative and absolute measures of association convey different information.^41,42^ Relative measures (eg, prevalence ratios) characterize the strength of association between interpersonal violence and handgun carrying; absolute measures (eg, prevalence differences) characterize excess prevalence of handgun carrying among those who have vs have not used interpersonal violence. Variation in relative measures could indicate distinct etiological characteristics associated with handgun carrying with implications for context-specific strategies to promote safety. Variation in absolute measures would provide information about the population burden of handgun carrying in association with interpersonal violence. Comprehensive surveillance of trends on both scales may lay the foundation for future etiological investigations and inform prevention resource allocation.

## Methods

This cross-sectional study was not considered human participants research by the University of Washington Human Subjects Division; therefore, the requirement for informed consent was waived. We followed the Strengthening the Reporting of Observational Studies in Epidemiology (STROBE) reporting guideline for cross-sectional studies.

### Data Sources

We used National Survey on Drug Use and Health (NSDUH) data for 2002 to 2019, made publicly available through the Substance Abuse and Mental Health Data Archive (SAMHDA) Restricted-use Data Analysis System.^43,44^ The NSDUH is an annual, nationally representative cross-sectional survey of the noninstitutionalized US civilian population ages 12 years and older.^45^ NSDUH uses stratified, multistage probability sampling. States and regions within states were stratified, and census tracts, census-block groups, and block-group segments were subsequently sampled with probability proportional to size. Dwelling units and individuals were then selected. NSDUH created weights to generate nationally representative estimates. Weighted interview response proportions were 65% to 79% across years.^45,46^ Additional details are available elsewhere.^45^

We used aggregate data from individuals aged 12 to 17 years. Publicly available individual-level data include a 3-level county classification of rural-urban but not the 6-level classification that is the focus of our study. Because our interest was in variation across the fullest possible spectrum of rural-urban status, we used aggregate data that include the 6-level rural-urban classification. SAMHDA combined data across 2-year intervals; we refer to intervals as years and reference them by the last year in the interval (eg, 2019 for 2018 to 2019).

### Measures

#### Demographic

Adolescents’ self-reported their sex (male or female) and race and ethnicity with NSDUH-defined options. Due to structural racism, racialized groups experience higher rates of firearm-related harms and may relate to firearms in unique ways.^7,47^ Therefore, describing the distribution of racial and ethnic groups is relevant for equity-centered interventions to prevent handgun carrying-related harms. To avoid suppressed cells, we used 4 race and ethnicity categories: Hispanic, non-Hispanic Black (hereafter, *Black*), non-Hispanic White (hereafter, *White*), and non-Hispanic other race or multiple races (including American Indian or Alaska Native, Asian, Native Hawaiian, other Pacific Islander, and other; beginning in 2013, Guamanian or Chamorro and Samoan were included).

#### County Rural-Urban Status

Adolescents’ county of residence was defined by the US Department of Agriculture’s 2003 Rural-Urban Continuum Codes 6-level classification.^48^ All counties in the US were divided into 1 of 6 categories: 3 metropolitan (ie, urban) counties, defined by metropolitan area resident population size (from the 2000 Census), and 3 nonmetropolitan (ie, rural) counties, defined by urbanization.^49^ The 6 categories are (1) large metro, population 1 000 000 or more; (2) small metro, population 250 000 to 1 000 000; (3) small metro, population fewer than 250 000; (4) nonmetro, 20 000 or more urban population; (5) nonmetro, 2500 to 19 999 urban population; and (6) nonmetro, fewer than 2500 urban population.

#### Interpersonal Violence

We measured adolescents’ past-year use of interpersonal violence with all 3 interpersonal violence-related questions asked of adolescents in the survey. The first pertained to serious fights: “During the past 12 months, how many times have you gotten into a serious fight at school or work?” The second pertained to group fights: “During the past 12 months, how many times have you taken part in a fight where a group of your friends fought against another group?” The last pertained to physical attacks with intent to harm: “During the past 12 months, how many times have you attacked someone with the intent to seriously hurt them?” All questions had response options 0, 1 to 2, 3 to 5, 6 to 9, and 10 or more times and were recoded to 1 or more vs none for parsimony. We analyzed questions separately.

#### Handgun Carrying

Adolescents were asked: “During the past 12 months, how many times have you carried a handgun?” Responses options were 0, 1 to 2, 3 to 5, 6 to 9, or 10 or more times. Given the low prevalence of frequent handgun carrying, responses were recoded to 1 or more vs none.

### Statistical Analysis

We estimated prevalence ratios (PRs) and prevalence differences (PDs) comparing handgun carrying prevalence among adolescents who did vs did not report interpersonal violence, separately by year and rural-urban status.

SAMHDA provided 95% CIs for survey-weighted prevalence estimates. These CIs were calculated with Taylor series linearization and incorporated NSDUH’s stratified, clustered design. To generate 95% CIs for contrasts of prevalence, ie, PRs and PDs, we used a Monte Carlo bootstrap procedure separately by year and rural-urban status. We first identified the lower and upper bounds of the SAMHDA-provided 95% CIs for handgun carrying prevalence among adolescents who did and did not use interpersonal violence. We then randomly sampled handgun carrying prevalence from a uniform distribution specified by these 95% CIs and calculated the PR and PD. We repeated this process 10 000 times and calculated 95% CIs for PRs and PDs by taking the 2.5% and 97.5% quantiles of the resulting distribution. Additional details and example code are in the eMethods in Supplement 1. This approach accounted for NSDUH’s weighting and complex survey design. The small amount of missing data (approximately 1%) were excluded from analyses.

Analyses were conducted in R statistical software version 4.0.0 (R Project for Statistical Computing). Data were analyzed from April to July 2022.

## Results

### Description of Study Sample

In each year, the sample included a weighted count of almost 25 million adolescents. For example, in 2019, the overall weighted count of adolescents was 24 900 000, with 50.9% (95% CI, 50.2%-51.6%) males and 13.5% (95% CI, 12.8%-14.2%) Black adolescents, 24.7% (95% CI, 23.8%-25.6%) Hispanic adolescents, and 51.8% (95% CI, 50.8%-52.8%) White adolescents (Table). Rural counties had higher percentages of White adolescents and smaller percentages of Black and Hispanic adolescents, eg, 81.1% (95% CI, 75.9%-85.4%) of adolescents were White in the most rural counties vs 43.1% (95% CI, 41.7%-44.6%) White adolescents in the most urban counties in 2019. Sample characteristics were similar across years.

**Table.** Description of Sample, US Adolescents National Survey on Drug Use and Health, 2018-2019

Characteristic	Individuals, No., thousands (%) [95% CI]^a^						
	Nonmetropolitan			Metropolitan			Total (N = 24 900 000)
	<2500 (n = 455 000)	2500-19 999 (n = 1 818 000)	≥20 000 (n = 1 646 000)	<250 000 (n = 2 393 000)	250 000-1 000 000 (n = 5 165 000)	>1 000 000 (n = 13 424 000)	
Sex							
Male	235 (51.6) [47.2-56.1]	909 (50.0) [47.9-52.1]	855 (52.0) [49.7-54.2]	1196 (50.0) [48.1-51.9]	2665 (51.6) [50.3-52.9]	6820 (50.8) [49.8-51.9]	12 680 (50.9) [50.2-51.6]
Female	220 (48.4) [43.9-52.8]	909 (50) [47.9-52.1]	791 (48) [45.8-50.3]	1197 (50) [48.1-51.9]	2500 (48.4) [47.1-49.7]	6605 (49.2) [48.1-50.2]	12 221 (49.1) [48.4-49.8]
Race and ethnicity							
Hispanic	26 (5.7) [3.5-9.4]	197 (10.8) [8.6-13.5]	288 (17.5) [14.7-20.8]	442 (18.5) [16.1-21.1]	1363 (26.4) [24.4-28.5]	3827 (28.5) [27.1-29.9]	6143 (24.7) [23.8-25.6]
Non-Hispanic Black	26 (5.7) [3.2-10.1]	171 (9.4) [7.5-11.6]	161 (9.8) [7.7-12.3]	276 (11.5) [9.9-13.4]	546 (10.6) [9.3-12.0]	2184 (16.3) [15.2-17.4]	3364 (13.5) [12.8-14.2]
Non-Hispanic White	369 (81.1) [75.9-85.4]	1342 (73.8) [70.6-76.8]	1076 (65.4) [62.0-68.6]	1533 (64.1) [61.3-66.7]	2784 (53.9) [51.8-56.0]	5791 (43.1) [41.7-44.6]	12 893 (51.8) [50.8-52.8]
Other race or multiple races, not Hispanic^b^	34 (7.5) [5.1-10.7]	109 (6.0) [5.0-7.2]	120 (7.3) [5.9-9.1]	142 (5.9) [5.0-7.0]	472 (9.1) [8.3-10.1]	1622 (12.1) [11.2-13.1]	2500 (10.0) [9.5-10.6]
Serious fighting^c^							
None	370 (81.4) [77.7-84.6]	1506 (82.8) [80.9-84.7]	1340 (81.5) [79.4-83.3]	1941 (81.1) [79.4-82.8]	4249 (82.3) [81.2-83.3]	11 131 (82.9) [82.1-83.7]	20 537 (82.5) [81.9-83.0]
≥1 times	80 (17.6) [14.4-21.4]	292 (16.1) [14.3-18.0]	284 (17.3) [15.5-19.2]	426 (17.8) [16.3-19.5]	864 (16.7) [15.7-17.8]	2164 (16.1) [15.4-16.9]	4112 (16.5) [16.0-17.0]
Attacked someone^c^							
None	424 (93.2) [90.0-95.5]	1740 (95.7) [94.8-96.5]	1559 (94.7) [93.5-95.7]	2266 (94.7) [93.7-95.6]	4901 (94.9) [94.3-95.5]	12 711 (94.7) [94.2-95.1]	23 602 (94.8) [94.5-95.1]
≥1 times	28 (6.1) [3.9-9.4]	72 (3.9) [3.2-4.9]	74 (4.5) [3.6-5.7]	109 (4.5) [3.8-5.5]	224 (4.3) [3.8-4.9]	604 (4.5) [4.1-4.9]	1111 (4.5) [4.2-4.8]
Group fighting^c^							
None	404 (88.9) [85.7-91.5]	1596 (87.8) [86.1-89.3]	1440 (87.5) [85.9-88.9]	2103 (87.9) [86.4-89.2]	4534 (87.8) [86.7-88.8]	11 865 (88.4) [87.7-89.0]	21 943 (88.1) [87.6-88.6]
≥1 times	46 (10.2) [7.6-13.4]	203 (11.2) [9.8-12.8]	185 (11.2) [9.9-12.7]	256 (10.7) [9.5-12.0]	580 (11.2) [10.2-12.3]	1418 (10.6) [9.9-11.2]	2688 (10.8) [10.4-11.3]
Handgun carrying^c^							
None	392 (86.2) [81.7-89.7]	1687 (92.8) [91.5-93.8]	1548 (94.1) [92.9-95.1]	2258 (94.4) [93.3-95.2]	4901 (94.9) [94.2-95.5]	12 819 (95.5) [95.0-95.9]	23 604 (94.8) [94.5-95.1]
≥1 times	56 (12.4) [8.9-16.9]	114 (6.3) [5.3-7.4]	82 (5.0) [4.1-6.0]	108 (4.5) [3.7-5.4]	219 (4.2) [3.7-4.9]	480 (3.6) [3.2-4.0]	1059 (4.3) [4.0-4.5]

^a^ Percentages and numbers are weighted. Results may not sum to column total due to missing data.

^b^ Includes American Indian or Alaska Native, Asian, Native Hawaiian, other Pacific Islander, Guamanian or Chamorro, Samoan, and a write-in option.

^c^ In the past 12 months.

Across counties in 2019, 16.5% (95% CI, 16.0%-17.0%) of adolescents reported past-year serious fighting, and 10.8% (95% CI, 10.4%-11.3%) reported past-year group fighting (Table). The prevalences of serious fighting and group fighting were similar across the 6 county types. A higher percentage of adolescents in nonmetropolitan counties with fewer than 2500 urban population reported attacking someone (6.1% [95% CI, 3.9%-9.4%] of adolescents) than other counties (ranging from 3.9%-4.5%). Interpersonal violence decreased overall from 2002 to 2019 (eFigure 1 in Supplement 1).

**Figure 1.**
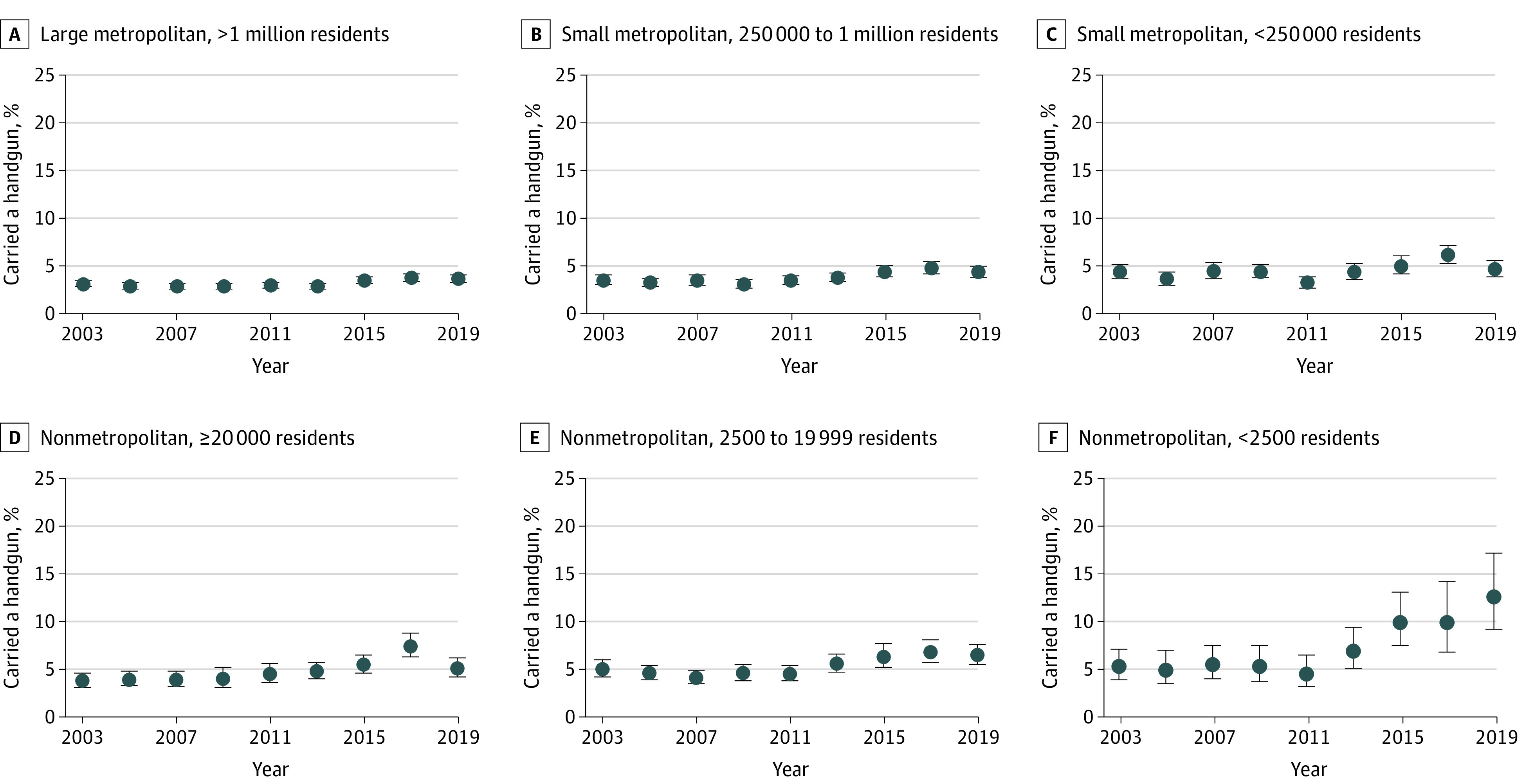
Prevalence of Past-Year Handgun Carrying Among US Adolescents by County Rural-Urban Status, 2002-2019 Bars reflect 95% CIs. Years are in 2-year intervals from 2002 to 2003 to 2018 to 2019.

Adolescent handgun carrying prevalence was higher with increasing rurality. In 2019, 12.4% (95% CI, 8.9%-16.9%) of adolescents in nonmetropolitan counties with fewer than 2500 urban population and 6.3% (95% CI, 5.3%-7.4%) of adolescents in nonmetropolitan counties with population 2500 to 19 999 reported past-year handgun carrying, compared with 3.6% to 5.0% in more urban counties (Table). Handgun carrying increased over time in all county types; the increase was especially pronounced in the most rural counties, where the prevalence of adolescent handgun carrying increased from 5.2% (95% CI, 3.8%-7.0%) in 2003 to 12.4% (95% CI, 8.9%-16.9%) in 2019. (Figure 1). Within rural-urban strata, handgun carrying was more common among adolescents who used interpersonal violence (eFigures 2-4 in Supplement 1).

### Association of Interpersonal Violence and Handgun Carrying Stratified by Rural-Urban Status

#### Prevalence Ratios

**Figure 2.**
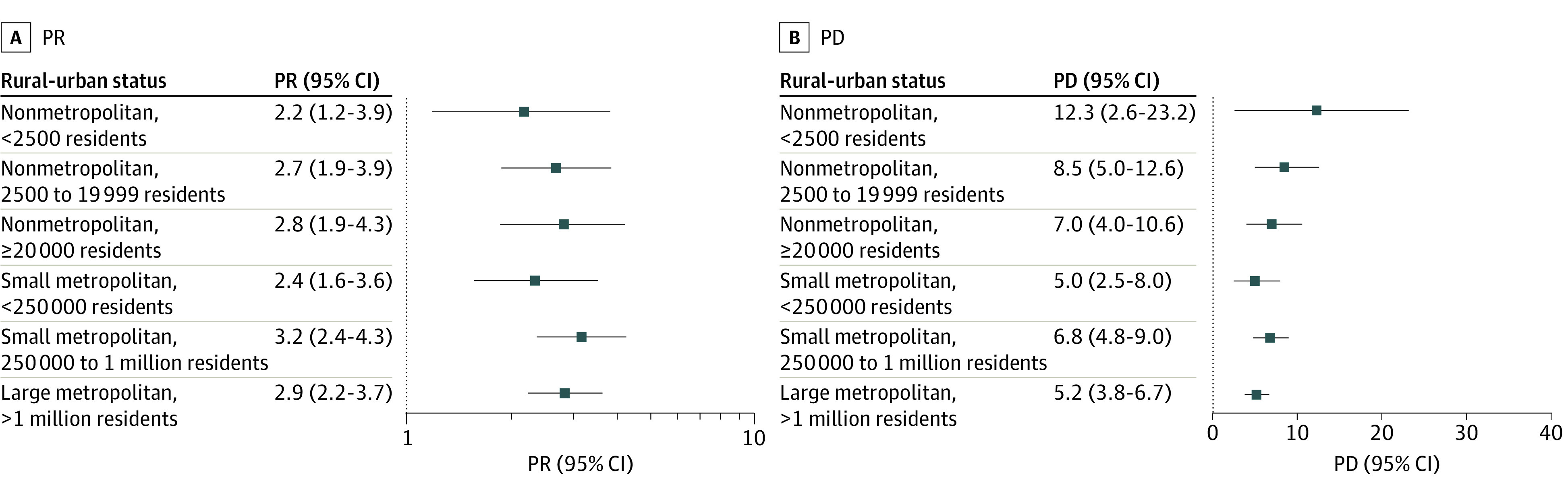
Association of Past-Year Adolescent Handgun Carrying and Serious Fighting, Stratified by Rural-Urban Status, 2018-2019 Prevalence ratios (PRs; untransformed) are plotted on log scale. PD indicates prevalence difference.

**Figure 3.**
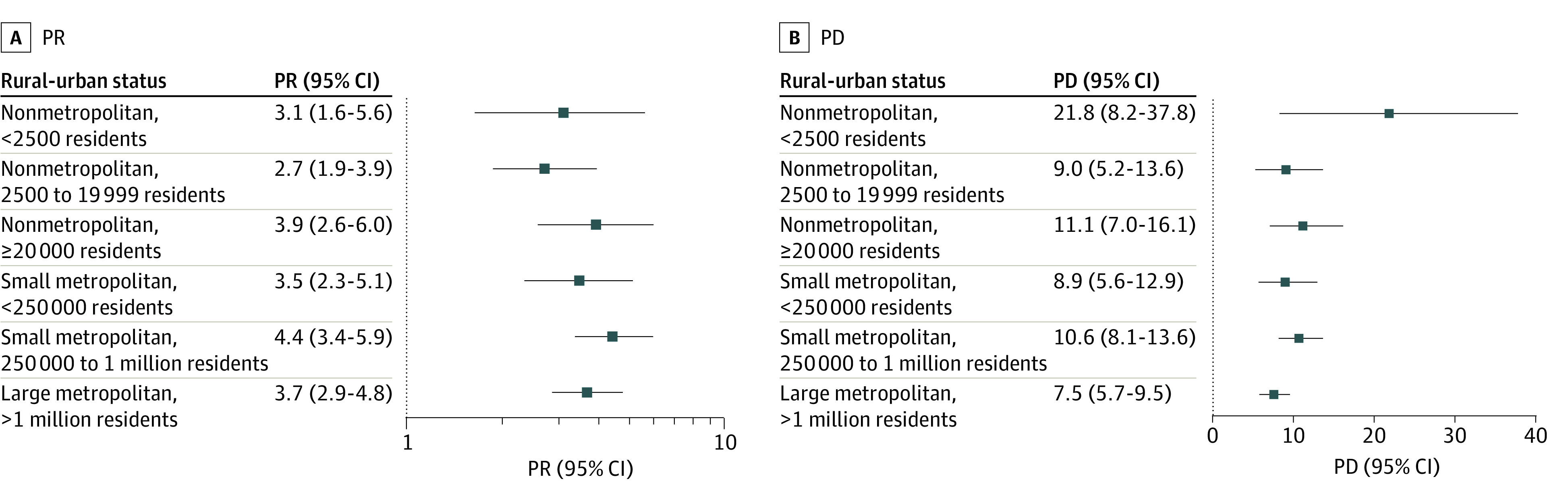
Association of Past-Year Adolescent Handgun Carrying and Group Fighting, Stratified by Rural-Urban Status, 2018-2019 Prevalence ratios (PRs; untransformed) are plotted on log scale. PD indicates prevalence difference.

**Figure 4.**
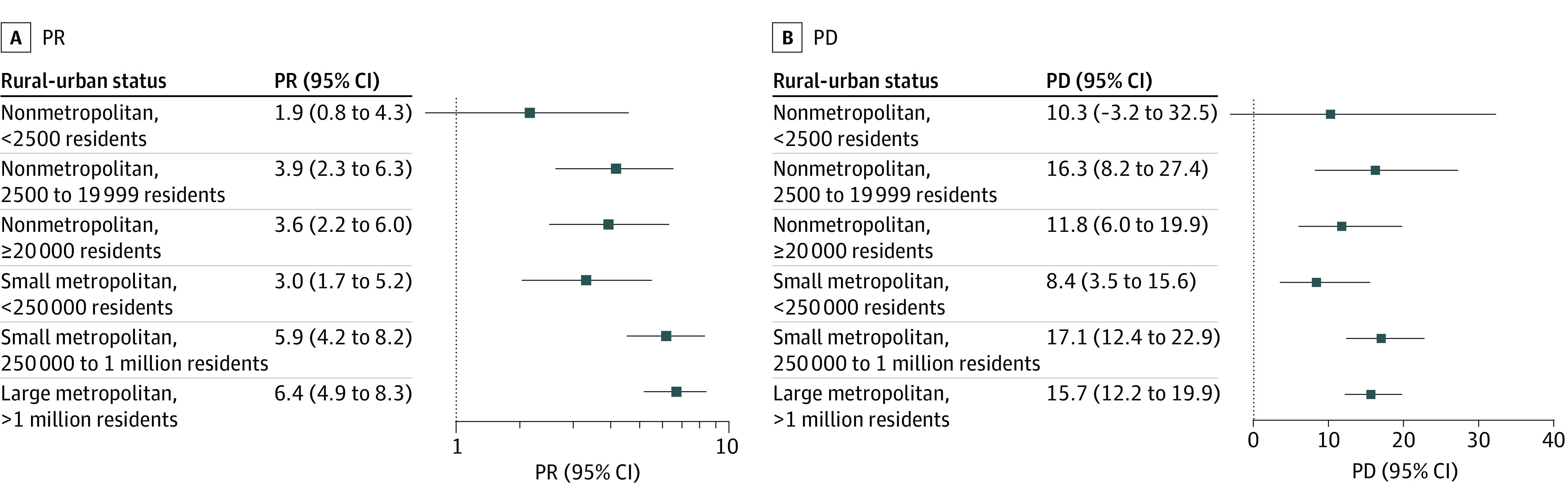
Association of Past-Year Adolescent Handgun Carrying and Attacking, Stratified by Rural-Urban Status, 2018-2019 Prevalence ratios (PRs; untransformed) are plotted on log scale. PD indicates prevalence difference.

In 2019, use of violence was associated with handgun carrying in all counties, with the highest prevalence ratio noted in association with attacking in the most urban counties (PR, 6.4% [95% CI, 4.9%-8.3%]) (Figure 2A, Figure 3A, and Figure 4A). For example, among adolescents in nonmetropolitan counties with fewer than 2500 urban population, handgun carrying prevalence was 2.2 (95% CI, 1.2-3.9) times greater among those who were vs were not in serious fight (Figure 2A). Among adolescents in the largest metropolitan counties, this PR was 2.9 (95% CI, 2.2-3.7). For group fighting, the PR was 3.1 (95% CI, 1.6-5.6) in the most rural counties and 3.7 (95% CI, 2.9-4.8) in the most urban counties (Figure 3A); while for attacking, the PR was 1.9 (95% CI, 0.8-4.3) in the most rural counties and 6.4 (95% CI, 4.9-8.3) in the most urban counties (Figure 4A). These patterns held across years (eFigures 5-7 in Supplement 1).

#### Prevalence Differences

In contrast to PRs, PDs showed less rural-urban variation and were, in some cases, larger in more rural counties (Figure 2B, Figure 3B, and Figure 4B). For example, in nonmetropolitan counties with fewer than 2500 urban population in 2019, the difference in handgun carrying prevalence comparing those who were vs were not in a serious fight was 12.3% (95% CI, 2.6%-23.2%) (Figure 2B). In the largest metropolitan counties, this PD was 5.2% (95% CI, 3.8%-6.7%). For group fighting, the PD was 21.8% (95% CI, 8.2%-37.8%) in the most rural counties and 7.5% (95% CI, 5.7%-9.5%) in the largest metropolitan counties (Figure 3B). For attacking, the PD was 10.3% (95% CI, −3.2%-32.5%) in the most rural counties and 15.7% (95% CI, 12.2%-19.9%) in the largest metropolitan counties (Figure 4B). Findings were consistent across years (eFigures 5-7 in Supplement 1).

## Discussion

This cross-sectional study found that across the rural-urban continuum, handgun carrying was substantially more common among adolescents who used interpersonal violence than among adolescents who did not. These findings are consistent with prior research among urban and national samples.^30^ In relative terms, prevalence ratios for the association between adolescent violence and handgun carrying were greater in urban areas than in rural areas. However, in absolute terms, due to the high prevalence of handgun carrying among rural adolescents, prevalence differences for that association were similar and sometimes greater in rural areas than in urban areas. That is, the greater overall prevalence of handgun carrying in rural areas resulted in a higher prevalence of adolescent handgun carrying associated with violence in rural areas than in urban areas. These patterns were consistent for multiple types of violence and across the study period.

Our findings indicate the importance of context in understanding adolescent handgun carrying and preventing its potential harms. Differences across socioecological levels may help account for the difference in the association in relative terms between handgun carrying and violence in rural vs urban areas in our study.^30^ In urban settings, adolescents who carry handguns have often been exposed to violence, carry for protection, and exhibit a constellation of risk behaviors (eg, fighting, using or selling drugs).^50,51,52,53,54^ In rural settings, social, cultural, and economic factors may increase perceived acceptability of adolescent handgun carrying and adolescents’ household handgun access, firearm training, and recreational and work-related handgun use.^13,18,21,22^ These factors could result in a higher overall prevalence of adolescent handgun carrying in rural areas and, in turn, make violence less likely to be a risk marker for handgun carrying.

Despite potential handgun use for recreation or socialization, adolescent handgun carrying nonetheless increases firearm-related injury risk.^30,31,55,56^ Our findings suggest that, in rural areas, interventions to prevent handgun-related harms should not focus only on adolescents who use interpersonal violence. Indeed handgun carrying prevalence among adolescents in the most rural counties who did not report interpersonal violence was similar to and sometimes higher than the prevalence among adolescents in more urban counties who did report interpersonal violence. If family and community members often govern adolescents’ access to and use of firearms in rural areas (eg, via household ownership, recreation, or work), they may be effective targets for interventions to promote adolescent firearm safety in these settings. For example, health care practitioners or other trusted messengers (eg, sport shooting instructors) could discuss handgun carrying along with other aspects of safer firearm use and storage with families and adolescents.^57^ School- or community-based programs that address the specific needs and cultures of rural communities, including firearm-related norms, may effectively prevent potential harms associated with adolescent handgun carrying. At the policy level, child access prevention laws, which hold parents liable for unsupervised firearm access, may also influence parental monitoring of adolescent firearm access.^58^ In urban contexts, tailored interventions might require a different focus, eg, reducing crime and community violence and therefore young people’s perceived need for self-protection.

While context-specific interventions to prevent handgun-related harms among adolescents may be useful, our findings suggest some interventions may be applicable in varied contexts. Indeed, because the absolute difference in handgun carrying prevalence between those who did vs did not use violence was at times greater in rural areas than in urban areas, strategies to prevent handgun carrying among those who use violence may benefit comparatively more adolescents in rural vs urban areas. This could be especially true today compared with 20 years ago; we found that adolescent handgun carrying increased in recent years, with the largest increases among adolescents in the most rural communities (nonmetropolitan counties with <2500 urban population) who used violence. For example, by 2019, one-third of adolescents in these counties who were in a group fight also carried a handgun. These findings align with evidence of overall increases and sociodemographic shifts in adolescent handgun carrying over time in the US.^34,39,59^ A 2022 study by Carey and Coley^34^ found that increases in adolescent handgun carrying from 2002 to 2019 were more pronounced among adolescents in rural vs urban areas. However, Carey and Coley^34^ defined urbanicity with a 3-level classification, with rural defined as fewer than 20 000 urban population. Our findings suggest variation in handgun carrying prevalence and correlates even within this rural category.

### Limitations

This study has some limitations. For ease of interpretation and precision of estimates, we used binary indicators of handgun carrying and violence; however, this resulted in some loss of information. Future research should examine variation in the frequency of these behaviors. CIs were sometimes wide, particularly for the most rural stratum. Cross-sectional data prevent us from determining which behaviors, interpersonal violence or handgun carrying, preceded the other. We had no information on handgun carrying motivations or the circumstances in which adolescents carried. Results may be influenced by misclassification, social desirability bias, or nonresponse bias; however, computer-assisted interviewing may reduce these biases. Aggregate data prevented individual-level covariate adjustment; however, our goal in this descriptive study was to characterize the association of interpersonal violence and handgun carrying, not estimate the effect of one on the other. Lastly, we focused exclusively on interpersonal violence; given the high incidence of suicide in rural areas and evidence that weapon carrying is associated with suicide risk behaviors,^55,56,60^ future research should examine adolescent handgun carrying in association with suicide risk across the rural-urban continuum.

## Conclusions

In this cross-sectional study, we identified differences in the associations between adolescent participation in interpersonal violence and handgun carrying across the rural-urban continuum, including among counties typically grouped into a general rural category. Sources of such variation should be studied further and considered when tailoring strategies to promote safety and reduce firearm-related harm.

## Notes

Supplement 1.
eMethods.
**eFigure 1.** Prevalence of Past-Year Interpersonal Violence Among US Adolescents (Aged 12-17 Years) by County Urban-Rural Status, 2002-2019 **eFigure 2.** Prevalence of Past-Year Handgun Carrying Among US Adolescents (Aged 12-17 Years) by County Urban-Rural Status and Past-Year Serious Fighting, 2002-2019 **eFigure 3.** Prevalence of Past-Year Handgun Carrying Among US Adolescents (Aged 12-17 Years) by County Urban-Rural Status and Past-Year Group Fighting, 2002-2019 **eFigure 4.** Prevalence of Past-Year Handgun Carrying Among US Adolescents (Aged 12-17 Years) by County Urban-Rural Status and Past-Year Attacking With Intent to Harm, 2002-2019 **eFigure 5.** Association of Past-Year Adolescent Handgun Carrying and Serious Fighting, Stratified by Rural-Urban Status, 2002-2017 **eFigure 6.** Association of Past-Year Adolescent Handgun Carrying and Group Fighting, Stratified by Rural-Urban Status, 2002-2017 **eFigure 7.** Association of Past-Year Adolescent Handgun Carrying and Attacking, Stratified by Rural-Urban Status, 2002-2017

Supplement 2.
Data Sharing Statement
