# Supplementary material for: Rural-Urban Variation in the Association of Adolescent Violence and Handgun Carrying in the United States, 2002-2019
Source: JAMA Netw Open. 2023 Feb 28;6(2):e231153. doi: 10.1001/jamanetworkopen.2023.1153 (PMC9975933; doi:10.1001/jamanetworkopen.2023.1153)
Supplement: Supplement 1. — eMethods. eFigure 1. Prevalence of Past-Year Interpersonal Violence Among US Adolescents (Aged 12-17 Years) by County Urban-Rural Status, 2002-2019 eFigure 2. Prevalence of Past-Year Handgun Carrying Among US Adolescents (Aged 12-17 Years) by County Urban-Rural Status and Past-Year Serious Fighting, 2002-2019 eFigure 3. Prevalence of Past-Year Handgun Carrying Among US Adolescents (Aged 12-17 Years) by County Urban-Rural Status and Past-Year Group Fighting, 2002-2019 eFigure 4. Prevalence of Past-Year Handgun Carrying Among US Adolescents (Aged 12-17 Years) by County Urban-Rural Status and Past-Year Attacking With Intent to Harm, 2002-2019 eFigure 5. Association of Past-Year Adolescent Handgun Carrying and Serious Fighting, Stratified by Rural-Urban Status, 2002-2017 eFigure 6. Association of Past-Year Adolescent Handgun Carrying and Group Fighting, Stratified by Rural-Urban Status, 2002-2017 eFigure 7. Association of Past-Year Adolescent Handgun Carrying and Attacking, Stratified by Rural-Urban Status, 2002-2017 [file jamanetwopen-e231153-s001.pdf]

Supplemental Online Content

Schleimer JP, Gause E, Dalve K, Ellyson A, Rowhani-Rahbar A. Rural-urban variation in the association of adolescent violence and handgun carrying in the United States, 2002-2019. *JAMA Netw Open*. 2023;6(3):e231153.
doi:10.1001/jamanetworkopen.2023.1153

eMethods.
eFigure 1. Prevalence of Past-Year Interpersonal Violence Among US Adolescents (Aged 12-17 Years) by County Urban-Rural Status, 2002-2019
eFigure 2. Prevalence of Past-Year Handgun Carrying Among US Adolescents (Aged 12-17 Years) by County Urban-Rural Status and Past-Year Serious Fighting, 2002-2019
eFigure 3. Prevalence of Past-Year Handgun Carrying Among US Adolescents (Aged 12-17 Years) by County Urban-Rural Status and Past-Year Group Fighting, 2002-2019
eFigure 4. Prevalence of Past-Year Handgun Carrying Among US Adolescents (Aged 12-17 Years) by County Urban-Rural Status and Past-Year Attacking With Intent to Harm, 2002-2019
eFigure 5. Association of Past-Year Adolescent Handgun Carrying and Serious Fighting, Stratified by Rural-Urban Status, 2002-2017
eFigure 6. Association of Past-Year Adolescent Handgun Carrying and Group Fighting, Stratified by Rural-Urban Status, 2002-2017
eFigure 7. Association of Past-Year Adolescent Handgun Carrying and Attacking, Stratified by Rural-Urban Status, 2002-2017

This supplemental material has been provided by the authors to give readers additional information about their work.

© 2023 Schleimer JP et al. *JAMA Network Open.*

eMethods.

Description of bootstrap procedure:

As described in the main text, SAMHDA provided 95% confidence intervals (CIs) for survey-weighted prevalence estimates. These CIs were calculated with Taylor series linearization and incorporated the NSDUH's stratified, clustered design. To generate 95% CIs for *contrasts* of prevalences, i.e., PRs and PDs, we used a Monte Carlo bootstrap procedure. This process was done separately by year and county rural-urban status. We first identified the lower and upper bounds of the SAMHDA-provided 95% CIs for handgun carrying prevalence among adolescents who did and did not use interpersonal violence. We then randomly sampled handgun carrying prevalence from a uniform distribution specified these 95% CIs (i.e., with ranges corresponding to the 95% CIs) and calculated the PR and PD. A uniform distribution assumes that any value within the range provided is equally likely; it is therefore more conservative than distributions which place more probability mass at the middle. As an example, the prevalence of handgun carrying in 2019 among adolescents who lived in large metro counties and who reported serious fighting was 8.0% (95% CI=6.7%-9.4%). The prevalence of handgun carrying in 2019 among adolescents who lived in large metro counties and who did not report serious fighting was 2.8% (95% CI=2.4%-3.2%). To generate a CI for the contrast between these estimates, we 1) randomly selected a number from a uniform distribution ranging from 6.7% to 9.4%, 2) randomly selected a number from a uniform distribution ranging from 2.4% to 3.2%, and 3) computed the contrast (i.e., ratio or difference). We repeated this process 10,000 times and calculated 95% CIs for PRs and PDs by taking the 2.5% and 97.5% quantiles of the resulting distribution.

Example Code:

```
## Generate confidence intervals for prevalence ratios and prevalence
differences from aggregate data that account for NSDUH survey design
and weighting

selectFromFun <- function(min, max) {
  select <- runif(10000, min=min, max=max)
}

for (c in 1:6) { # 6 county types
    for (y in seq(from=2003, to=2019, by=2)) {
        old <- Sys.time()
        print(paste0("year: ",y,", county: ",c))
     # within county type and year, compare proportion who carried
handgun among those who got in a serious fight vs. those who never got
in serious fight
      point <- df[df$year==y & df$county==c &
df$YOUTH.HAD.SERIOUS.FIGHT.AT.SCHOOL.WORK=="1 - One or More
Times"]$Column.. # Point estimate HG carrying prevalence among those
who got in serious fight 1+ times
      print(point)
```

© 2023 Schleimer JP et al. *JAMA Network Open.*

```
      min <- df[df$year==y & df$county==c & 
df$YOUTH.HAD.SERIOUS.FIGHT.AT.SCHOOL.WORK=="1 - One or More 
Times"]$Column...CI..lower. # Lower bound 95% CI HG carrying 
prevalence among those who got in serious fight 1+ times
      print(min)
      max <- df[df$year==y & df$county==c & 
df$YOUTH.HAD.SERIOUS.FIGHT.AT.SCHOOL.WORK=="1 - One or More 
Times"]$Column...CI..upper. # Upper bound 95% CI HG carrying 
prevalence among those who got in serious fight 1+ times
      print(max)

     dist <-  rep(NA,10000)
         for (i in 1:10000) {
          dist[i] <-  sample(selectFromFun(min,max),1,replace = T) # 
sample from uniform distribution based on lower and upper bounds
         }

     # reference: never got in fight
     refPoint <-  df[df$year==y & df$county==c & 
df$YOUTH.HAD.SERIOUS.FIGHT.AT.SCHOOL.WORK=="2 - None"]$Column.. # 
Point estimate HG carrying prevalence among those who never got in 
serious fight
     refMin <- df[df$year==y & df$county==c & 
df$YOUTH.HAD.SERIOUS.FIGHT.AT.SCHOOL.WORK=="2 - 
None"]$Column...CI..lower. # Lower bound 95% CI HG carrying prevalence 
among those who never got in serious fight
     refMax <- df[df$year==y & df$county==c & 
df$YOUTH.HAD.SERIOUS.FIGHT.AT.SCHOOL.WORK=="2 - 
None"]$Column...CI..upper. # Upper bound 95% CI HG carrying prevalence 
among those who never got in serious fight

     refDist <-  rep(NA,10000)
         for (i in 1:10000) {
          refDist[i] <-  sample(selectFromFun(refMin,refMax),1,replace 
= T) # sample from uniform distribution based on lower and upper 
bounds
         }

     # prevalence ratio
     PR <-  round(point/refPoint,3)
     PR_CI <-  round(quantile(dist/refDist,c(0.025,0.975)),3)
     result_ratio <- 
as.data.frame(cbind(PR=PR,PR_lb=PR_CI[1],PR_ub=PR_CI[2]))

     # prevalence difference
     PD <-  round(point-refPoint,3)
```

© 2023 Schleimer JP et al. *JAMA Network Open.*

```
    PD_CI <-  round(quantile(dist-refDist,c(0.025,0.975)),3)
    result_diff <-
as.data.frame(cbind(PD=PD,PD_lb=PD_CI[1],PD_ub=PD_CI[2]))

    # combine
    result <- cbind(result_ratio,result_diff)
    result$year <- y
    result$comparison <- c

    # assign name
    assign(paste0("result",c,"_",y) , result)
     new <- Sys.time() - old
     print(new)
   }
  }
```

© 2023 Schleimer JP et al. *JAMA Network Open.*

eFigure 1. Prevalence of Past-Year Interpersonal Violence Among US Adolescents (Aged 12-17 Years) by County Urban-Rural Status, 2002-2019

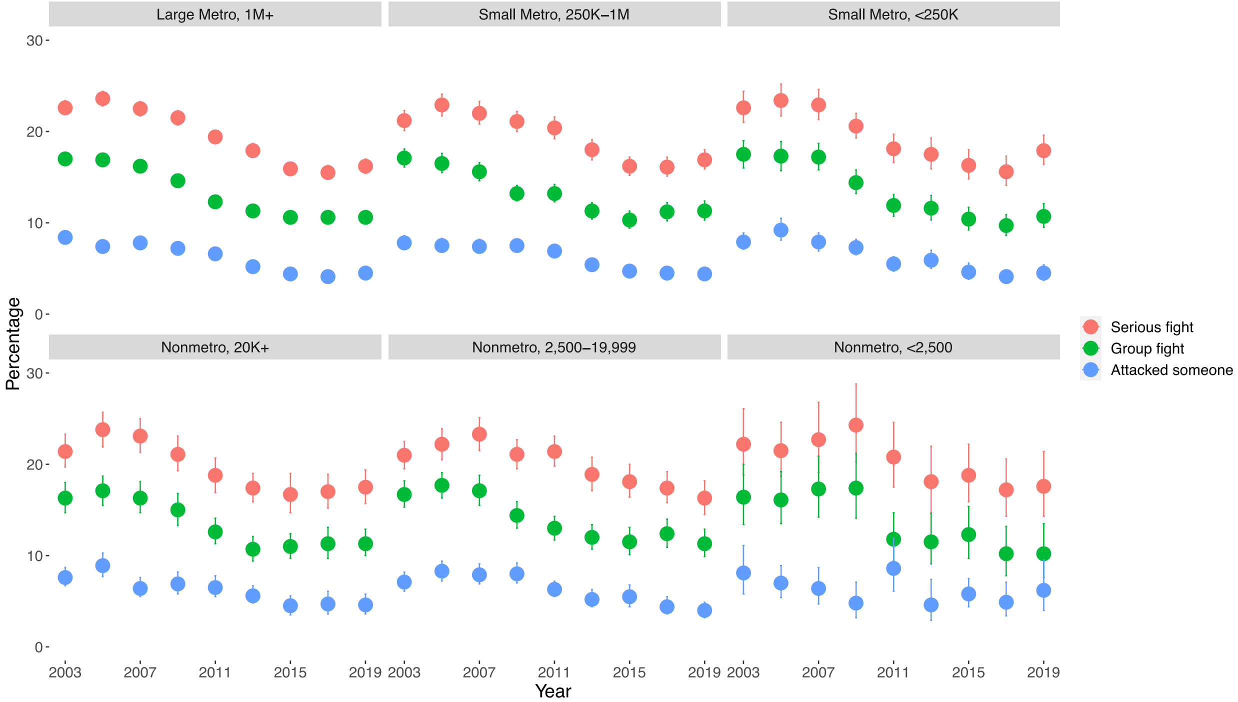

Note. Bars reflect 95% CI intervals. Years are in two-year intervals from 2002-2003 to 2018-2019.

© 2023 Schleimer JP et al. *JAMA Network Open.*

eFigure 2. Prevalence of Past-Year Handgun Carrying Among US Adolescents (Aged 12-17 Years) by County Urban-Rural Status and Past-Year Serious Fighting, 2002-2019

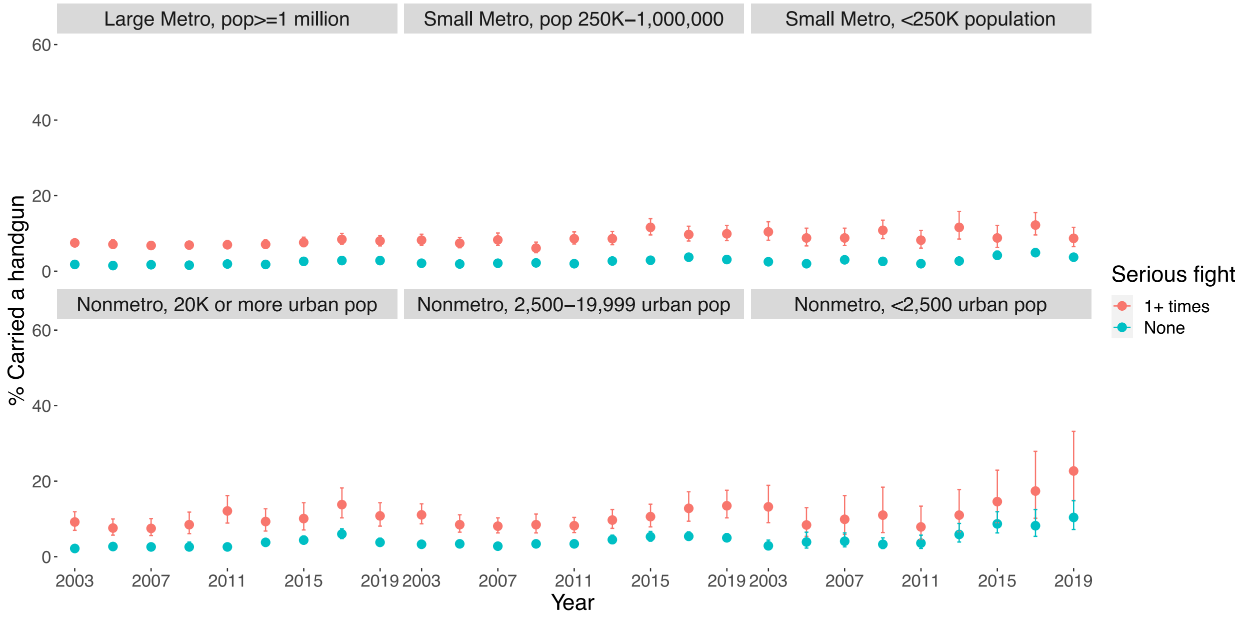

Note. Bars reflect 95% CI intervals. Years are in two-year intervals from 2002-2003 to 2018-2019.

© 2023 Schleimer JP et al. *JAMA Network Open.*

eFigure 3. Prevalence of Past-Year Handgun Carrying Among US Adolescents (Aged 12-17 Years) by County Urban-Rural Status and Past-Year Group Fighting, 2002-2019

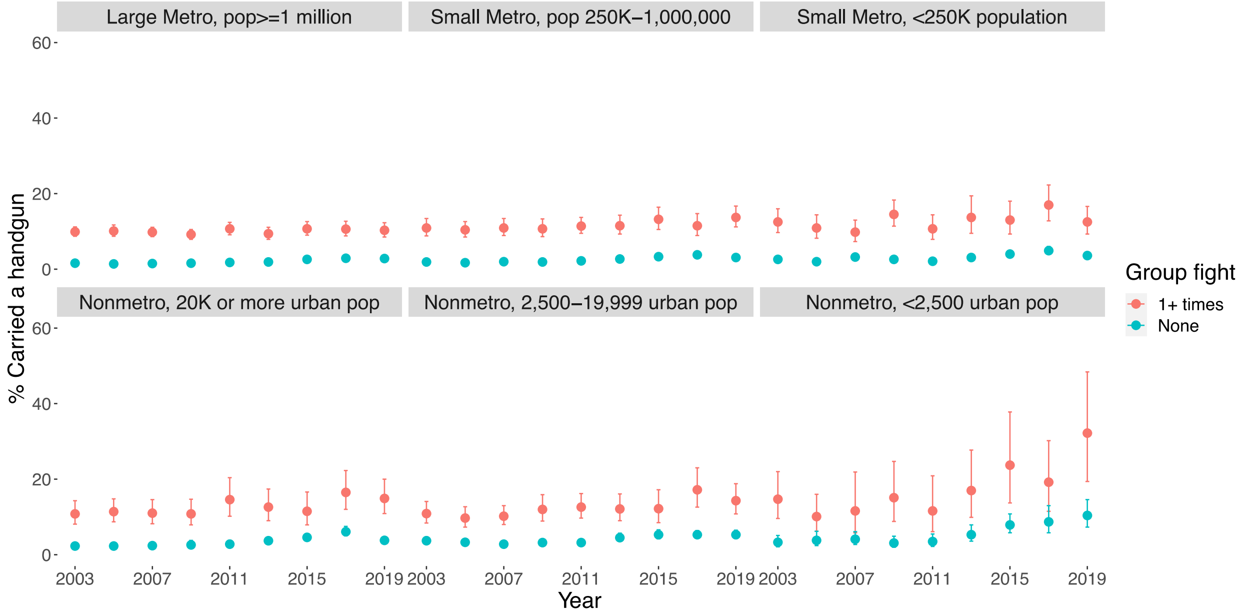

Note. Bars reflect 95% CI intervals. Years are in two-year intervals from 2002-2003 to 2018-2019.

© 2023 Schleimer JP et al. *JAMA Network Open.*

eFigure 4. Prevalence of Past-Year Handgun Carrying Among US Adolescents (Aged 12-17 Years) by County Urban-Rural Status and Past-Year Attacking With Intent to Harm, 2002-2019

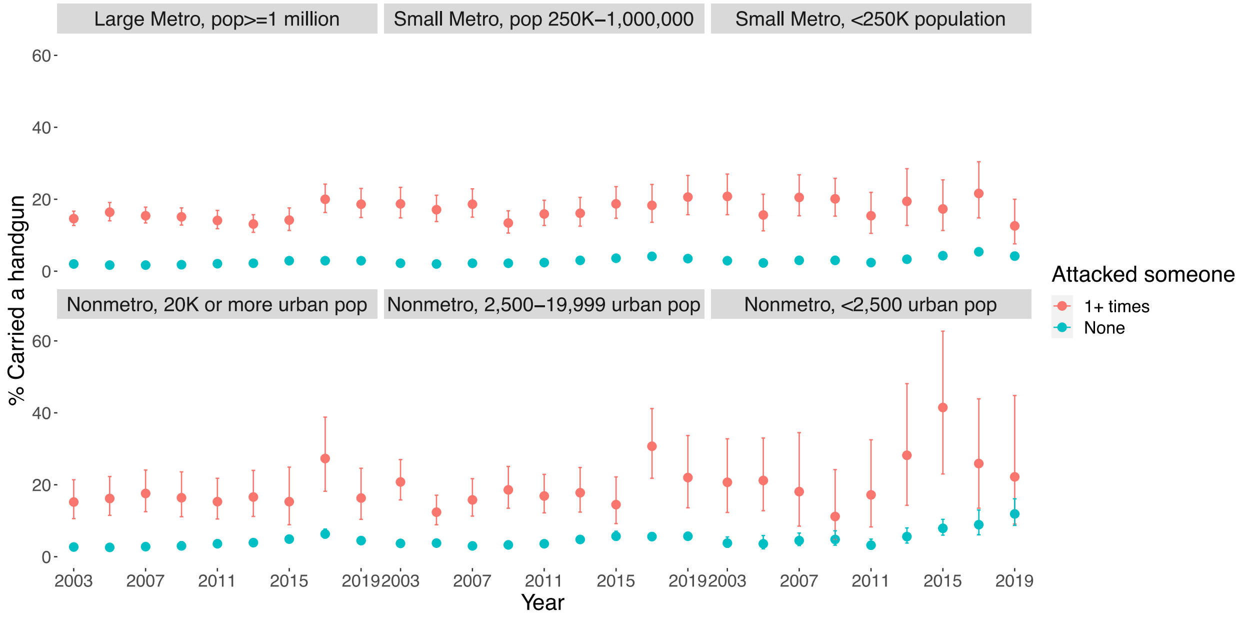

Note. Bars reflect 95% CI intervals. Years are in two-year intervals from 2002-2003 to 2018-2019.

© 2023 Schleimer JP et al. *JAMA Network Open.*

eFigure 5. Association of Past-Year Adolescent Handgun Carrying and Serious Fighting, Stratified by Rural-Urban Status, 2002-2017

A) Prevalence Ratios and 95% CIs

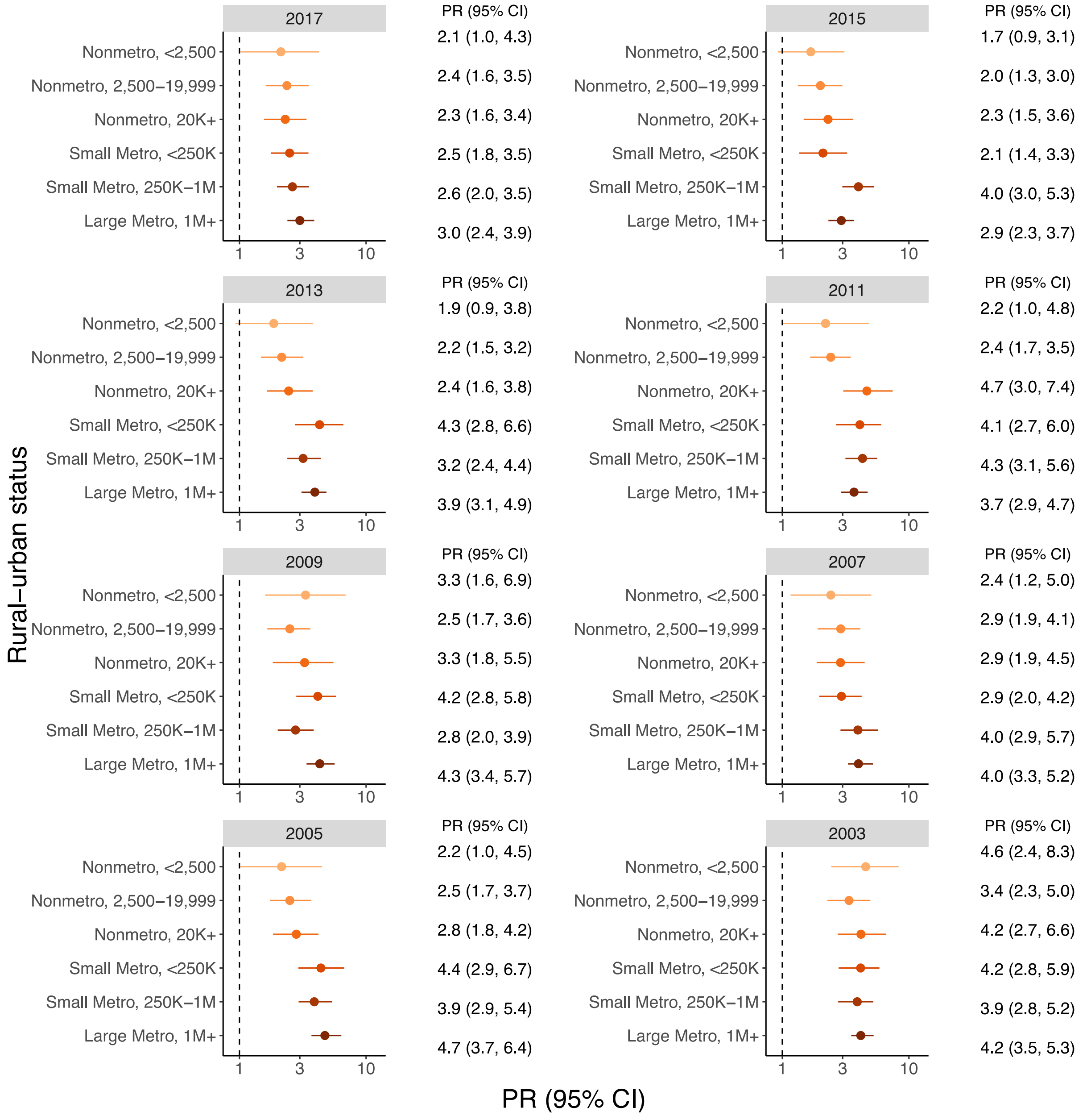

© 2023 Schleimer JP et al. *JAMA Network Open.*

B) Prevalence Differences and 95% CIs

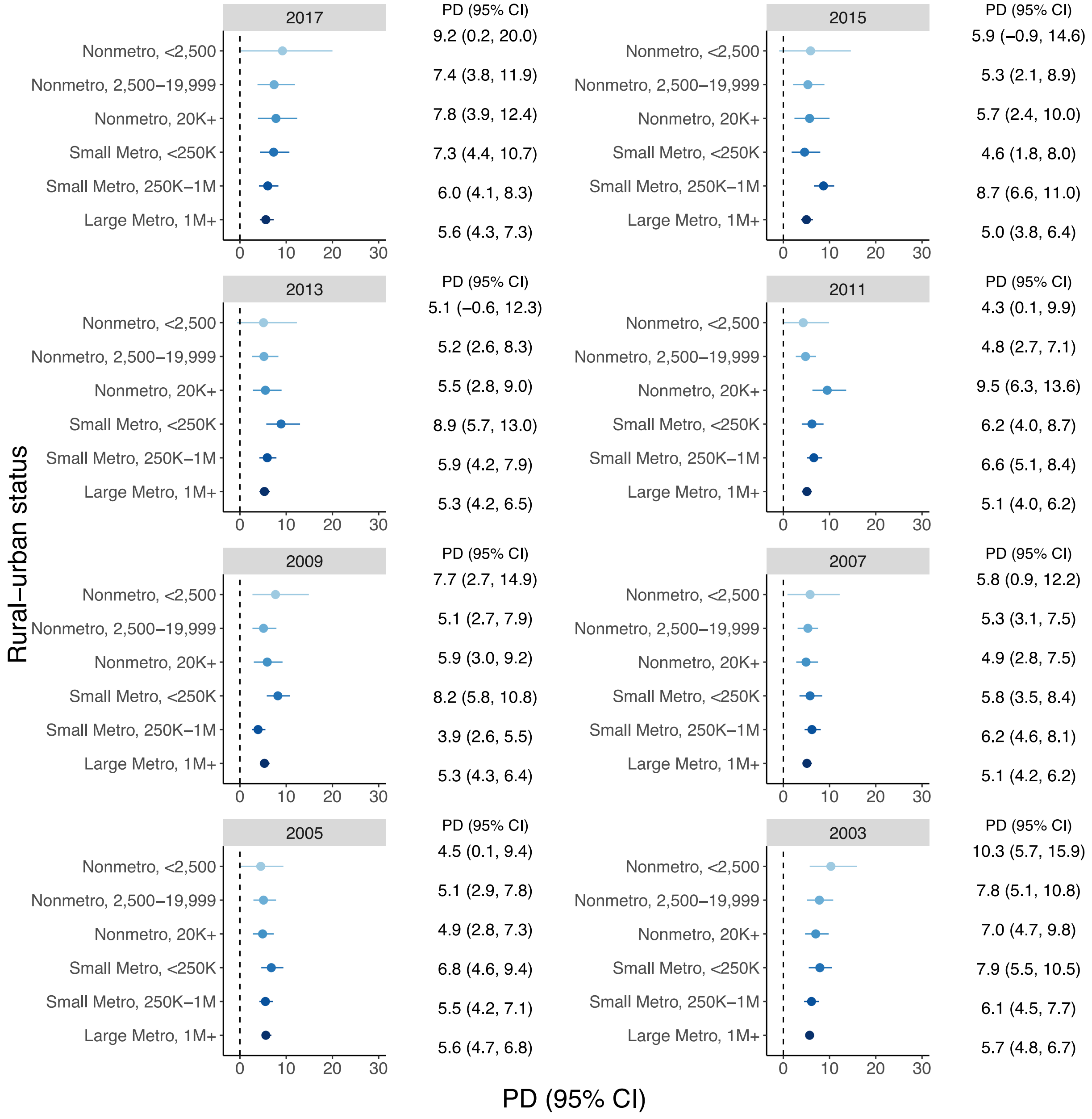

Note. Prevalence ratios (untransformed) are plotted on log scale. Years are in two-year intervals from 2002-2003 to 2016-2017.

© 2023 Schleimer JP et al. *JAMA Network Open.*

eFigure 6. Association of Past-Year Adolescent Handgun Carrying and Group Fighting, Stratified by Rural-Urban Status, 2002-2017

A) Prevalence Ratios and 95% CIs

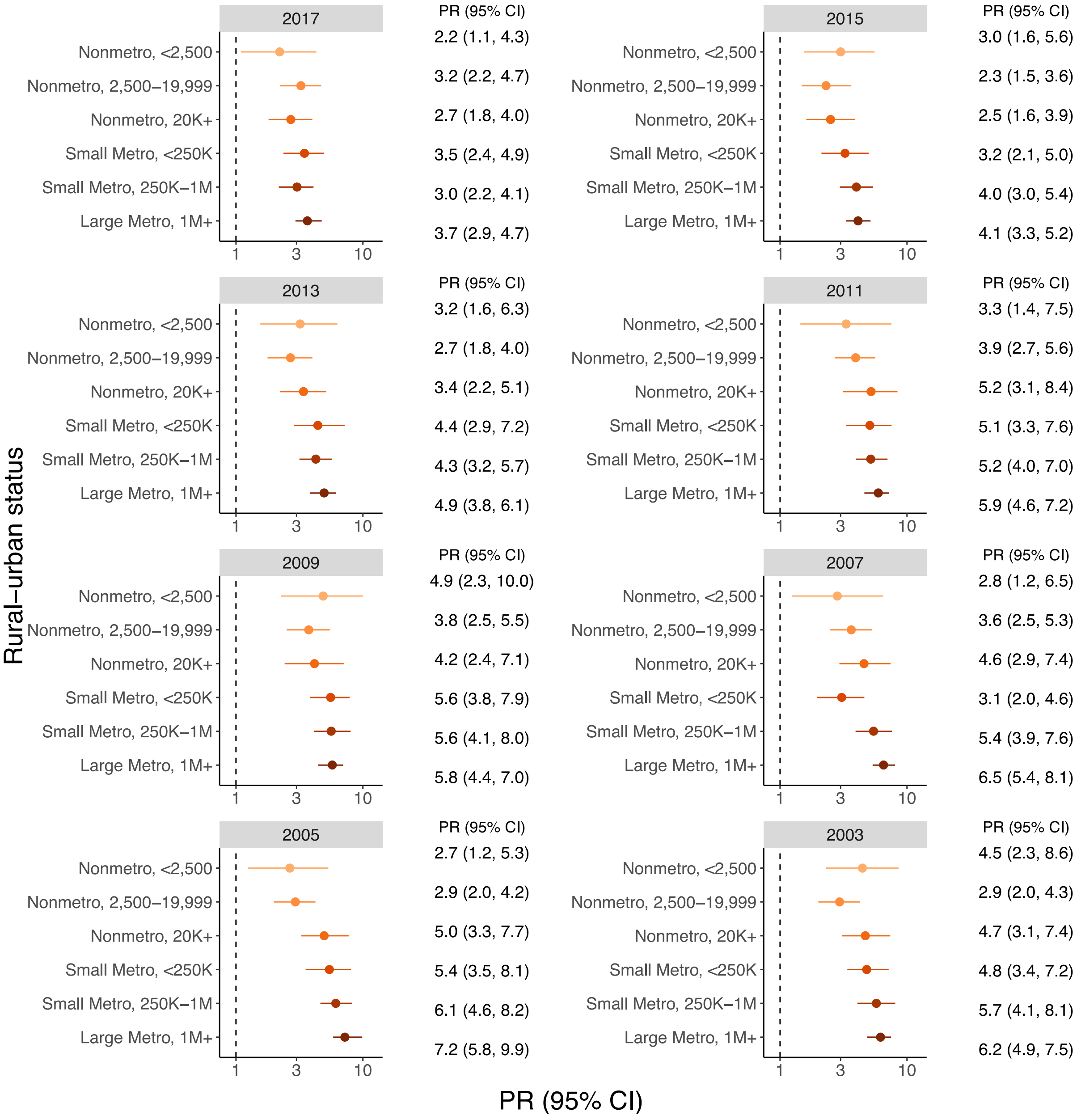

© 2023 Schleimer JP et al. *JAMA Network Open.*

B) Prevalence Differences and 95% CIs

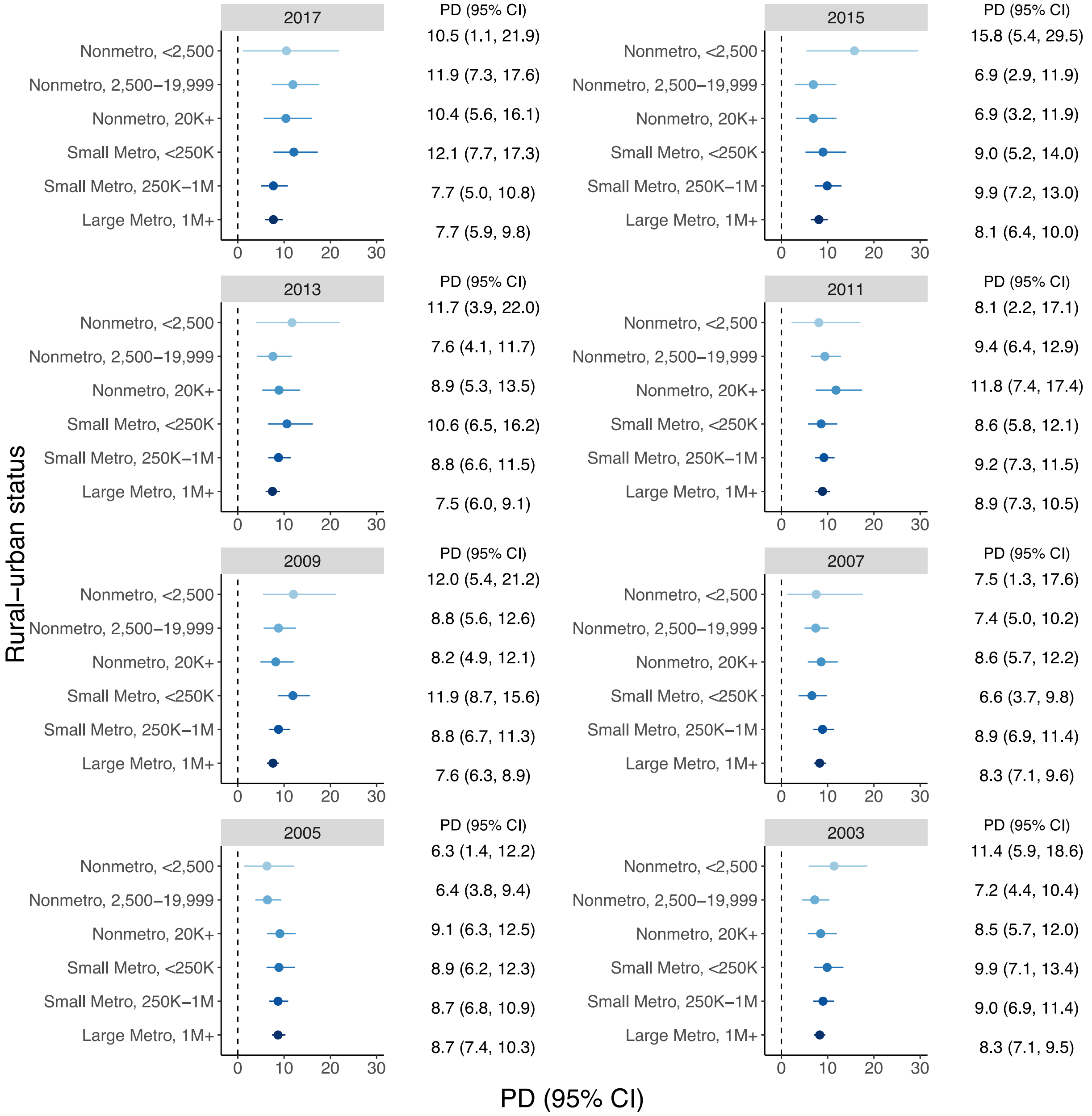

Note. Prevalence ratios (untransformed) are plotted on log scale. Years are in two-year intervals from 2002-2003 to 2016-2017.

© 2023 Schleimer JP et al. *JAMA Network Open.*

eFigure 7. Association of Past-Year Adolescent Handgun Carrying and Attacking, Stratified by Rural-Urban Status, 2002-2017

A) Prevalence Ratios and 95% CIs

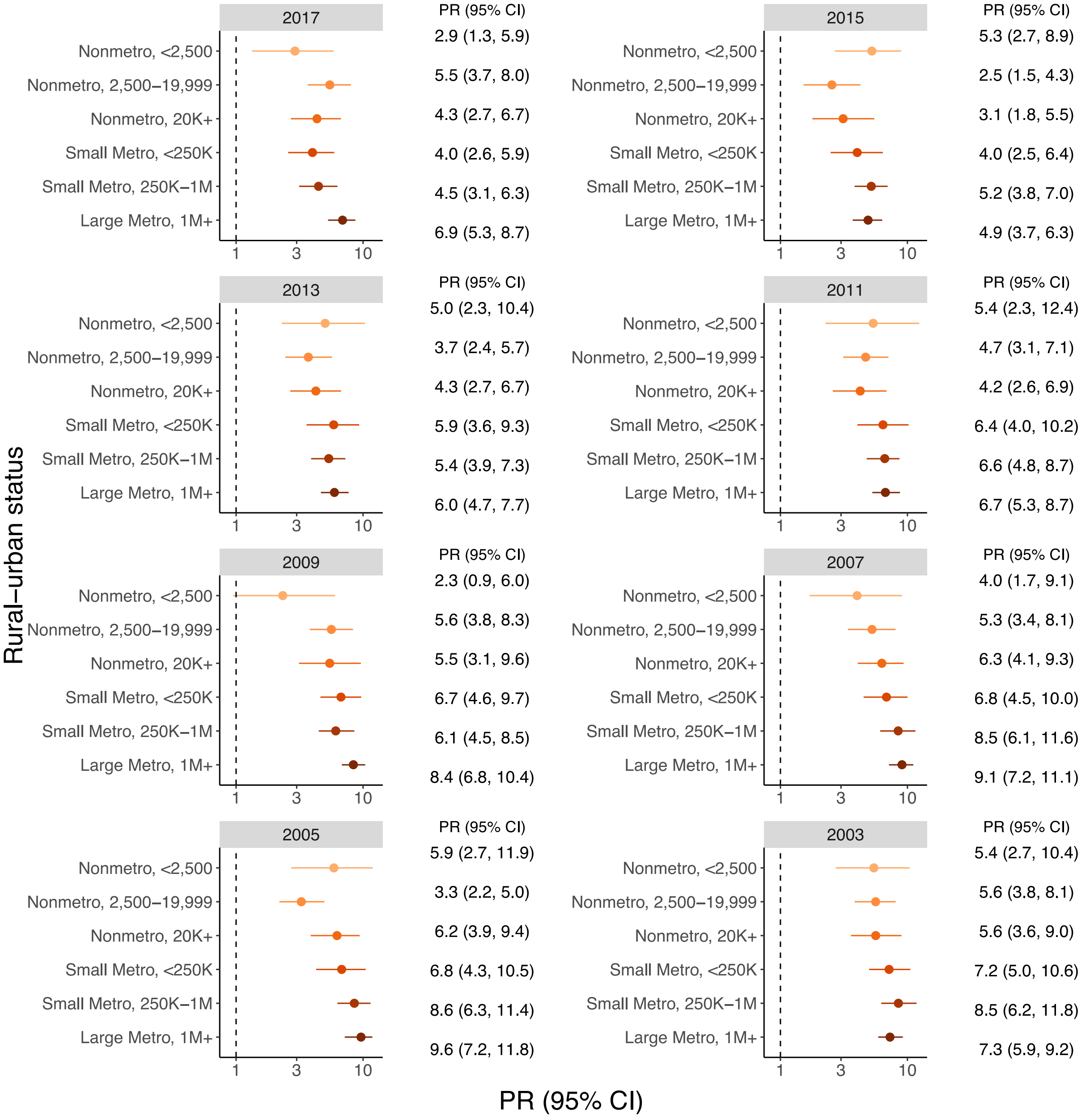

© 2023 Schleimer JP et al. *JAMA Network Open.*

B) Prevalence Differences and 95% CIs

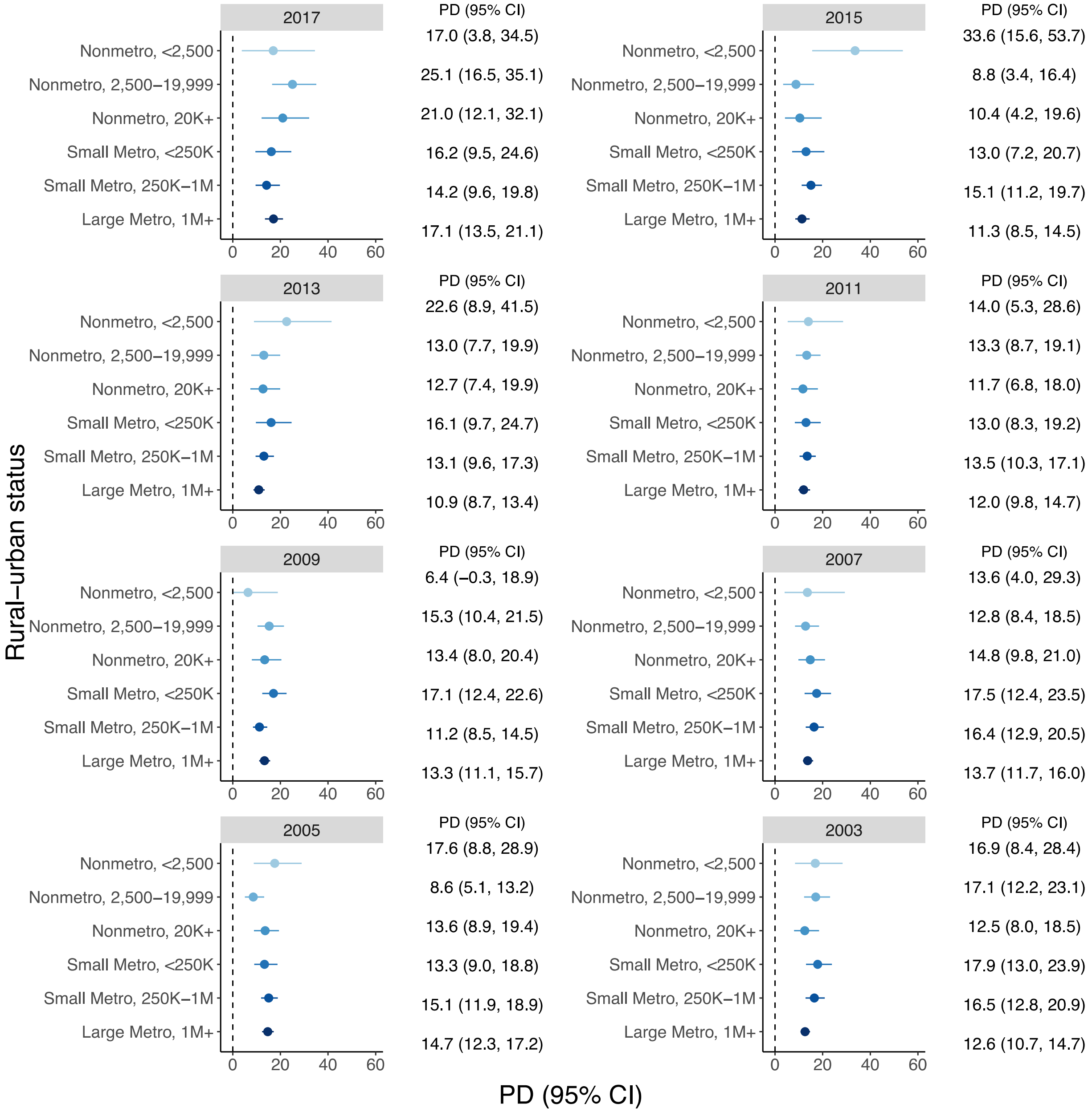

Note. Prevalence ratios (untransformed) are plotted on log scale. Years are in two-year intervals from 2002-2003 to 2016-2017.

© 2023 Schleimer JP et al. *JAMA Network Open.*
