# Supplementary material for: Rural-Urban Variation in the Association of Adolescent Violence and Handgun Carrying in the United States, 2002-2019
Source: JAMA Netw Open. 2023 Feb 28;6(2):e231153. doi: 10.1001/jamanetworkopen.2023.1153 (PMC9975933; doi:10.1001/jamanetworkopen.2023.1153)
Supplement: Supplement 2. — Data Sharing Statement [file jamanetwopen-e231153-s002.pdf]

Data Sharing Statement

Schleimer. Rural-Urban Variation in the Association of Adolescent Violence and Handgun Carrying in the United States, 2002-2019. *JAMA Netw Open*. Published February 28, 2023. doi:10.1001/jamanetworkopen.2023.1153

Data
Data available: No

Additional Information
Explanation for why data not available: Data are publicly-available.
